# A male infant with eczema and persistent thrombocytopenia, without micro-platelets: an atypical Wiskott-Aldrich syndrome?

**DOI:** 10.1186/1939-4551-8-S1-A148

**Published:** 2015-04-08

**Authors:** Paula Danielle Santa Maria De Albuquerque, Juliana Letícia Poli, Maria Eduarda Pontes Cunha De Castro, Persio Roxo Jr

**Affiliations:** 1 Ribeirão Preto Medical School, University of São Paulo, Brazil

## Background

Wiskott–Aldrich syndrome (WAS) is a rare X-linked recessive disorder characterized by early micro-thrombocytopenia, eczematous skin disease and recurrent infections. The syndrome is caused by mutations in gene *WAS* which codes WASP protein, that is expressed selectively in hematopoietic cells and it is involved in cell signaling and cytoskeleton reorganization. Micro-thrombocytopenia is the key hematological finding in patients with WAS. However, a normal mean platelet volume or the presence of giant platelets do not exclude a diagnosis of WAS.

## Methods

We describe a male infant, one month and sixteen days of life, that presented severe thrombocytopenia (< 70.000/mm^3^ without morphological changes), petechiae and purpura since birth. On the third day of life, the patient presented eczematous lesions on the trunk and face. The patient remained well during the follow-up. Other possible causes of neonatal thrombocytopenia associated with skin lesions like congenital infections, neonatal lupus and onco-hematological diseases were excluded. On the 63th day of life, he presented the first skin infection; thereafter there were two more skin infections, and a third episode of infection that compromised the central nervous system, evidenced by seizures (bleeding was excluded by computerized tomography). He presented worsening of breathing pattern and oxygen dependency, without apparent cause, even after improvement of the infection. At four months of life, he developed respiratory failure and death.

## Results

Hematological analyses: persistent thrombocytopenia since birth. Bone marrow was normal. Negative serology for congenital infections. Serum levels of IgG and IgA were normal, IgM was low. IgE was 71 kU/L. Genetic analysis for mutation of *WAS* gene is ongoing.

## Conclusions

The presence of early persistent thrombocytopenia with small platelets is a strong indicator of WAS. However, the absence of platelet volume changes does not exclude the diagnosis. Clinical signs must be considered for the diagnosis suspected of this rare and severe disease.

